# The experience of sensorimotor integration of a lower limb sensory neuroprosthesis: A qualitative case study

**DOI:** 10.3389/fnhum.2022.1074033

**Published:** 2023-01-11

**Authors:** Melissa S. Schmitt, John D. Wright, Ronald J. Triolo, Hamid Charkhkar, Emily L. Graczyk

**Affiliations:** ^1^Louis Stokes Cleveland Department of Veterans Affairs Medical Center, Cleveland, OH, United States; ^2^Frances Payne Bolton School of Nursing, Case Western Reserve University, Cleveland, OH, United States; ^3^Department of Biomedical Engineering, Case Western Reserve University, Cleveland, OH, United States

**Keywords:** sensory neuroprosthesis, amputation rehabilitation, home use, lower limb prosthesis, sensorimotor learning, phantom limb, sensory feedback, prosthesis embodiment

## Abstract

**Introduction:**

Lower limb prosthesis users often struggle to navigate uneven terrain or ambulate in low light conditions where it can be challenging to rely on visual cues for balance and walking. Sensory feedback about foot-floor interactions may allow users to reduce reliance on secondary sensory cues and improve confidence and speed when navigating difficult terrain. Our group has developed a Sensory Neuroprosthesis (SNP) to restore sensation to people with lower limb amputation by pairing electrical stimulation of nerves in the residual limb applied *via* implanted neurotechnology with pressure sensors in the insole of a standard prosthesis. Stimulation applied to the nerves evoked sensations perceived as originating on the missing leg and foot.

**Methods:**

This qualitative case study reports on the experiences of a 68-year-old with a unilateral trans-tibial amputation who autonomously used the SNP at home for 31 weeks. Interview data collected throughout the study period was analyzed using a grounded theory approach with constant comparative methods to understand his experience with this novel technology and its impacts on his daily life.

**Results:**

A conceptual model was developed that explained the experience of integrating SNP-provided sensory feedback into his body and motor plans. The model described the requirements of integration, which were a combination of a low level of mental focus and low stimulation levels. While higher levels of stimulation and focus could result in distinct sensory percepts and various phantom limb experiences, optimal integration was associated with SNP-evoked sensation that was not readily perceivable. Successful sensorimotor integration of the SNP resulted in improvements to locomotion, a return to a more normal state, an enhancement of perceived prosthesis utility, and a positive outlook on the experience.

**Discussion:**

These outcomes emerged over the course of the nearly 8 month study, suggesting that findings from long-term home studies of SNPs may differ from those of short-term in-laboratory tests. Our findings on the experience of sensorimotor integration of the SNP have implications for the optimal training of SNP users and the future deployment of clinical SNP systems for long-term home use.

## Introduction

Despite advances in commercially available prosthetic devices, lower limb amputees still suffer from certain functional deficits and often adopt an uneven gait that favors the intact leg (Prinsen et al., 2011). A recent review demonstrated that over-reliance on the intact leg in people with unilateral lower limb loss significantly decreased bone density and increased muscle atrophy in the residual limb (Finco et al., 2022). The loss of muscle strength, in addition to balance instability, leads to a higher rate of falls (Steinberg et al., 2018), and the loss of bone increases the likelihood of fall-related fractures (Haleem et al., 2021). Further, reliance on the intact leg leads to overuse injuries such as osteoarthritis (Lloyd et al., 2010), chronic low back pain (Kulkarni et al., 2005), and lumbar spine injuries (Farrokhi et al., 2018). Pain and uneven gait can cause additional complications, such as decreased balance confidence (Miller et al., 2002) and reduced engagement in social activities (Miller and Deathe, 2011; Sions et al., 2020).

Providing sensory feedback about foot-floor interactions is one potential means of restoring confidence in balance, improving gait symmetry, reducing reliance on the intact limb, and increasing prosthesis usage for lower limb prosthesis users. While sensory feedback can be conveyed through non-invasive sensory substitution methods such as tactors placed in the prosthesis socket (Crea et al., 2015; Rokhmanova and Rombokas, 2019; Escamilla-Nunez et al., 2020), one promising method of restoring naturalistic sensation is *via* peripheral nerve stimulation through chronically-implanted neural interfaces. Peripheral nerve stimulation to restore sensation has been tested in both upper and lower limb amputees (Clark et al., 2014; Ortiz-Catalan et al., 2014; Raspopovic et al., 2014; Tan et al., 2014; Charkhkar et al., 2018; Petrini et al., 2019), and it has been shown to produce sensation perceived as originating from the missing limb reliably and stably for more than 10 years (Tan et al., 2015). Sensations evoked by stimulation can then be paired with an instrumented prosthetic limb to create a Sensory Neuroprosthesis (SNP) for functional use. In laboratory testing, the sensation provided by SNPs to lower limb prosthesis users have resulted in significant reductions in postural sway (Charkhkar et al., 2020), improvements in ambulatory searching (Christie et al., 2020), improved phantom experience (Charkhkar et al., 2018; Petrini et al., 2019), and decreases in metabolic cost (Petrini et al., 2019).

For lower limb SNP technology to be successfully translated, the usability and user experience of the technology must be understood from the point of view of actual users (Hamilton and Finley, 2019). This information can lead to technology improvements aligning with user needs. Conducting interviews that allow the user to describe the experience and performing qualitative analysis on interview data is a holistic way to assess user experience. Prior qualitative studies have illustrated the experiences of lower limb amputees related to phantom limb pain (Camacho et al., 2021), prosthesis decision-making (Resnik et al., 2019), psychological adjustment (Abouammoh et al., 2021), work participation (Stuckey et al., 2020), and embodiment (Murray, 2004). However, no qualitative studies have described the impact of restored sensation with an SNP on prosthesis experience for lower limb amputees. This home study enabled the participant to engage in personally-meaningful tasks outside of a supervised laboratory environment, and the study length provided the participant an opportunity to reflect on his experiences over time. Prior qualitative studies with upper limb SNP devices used in the home have shed light on important user experiences, such as naturalness (Cuberovic et al., 2019; Graczyk et al., 2019) and phantom limb experience (Cuberovic et al., 2019; Lendaro et al., 2020). In this study, we collected interview data from a participant with unilateral transtibial limb loss over a total of 9.5 months while he used an SNP in his home and community. We employed grounded theory methods to assess the participant’s perspectives and opinions. The purpose of this qualitative case study was to understand and describe the experience of extended periods of home use of an SNP by a lower limb amputee. The theoretical model developed through this analysis identified concepts and experiences important to a lower limb SNP user and will be used to generate hypotheses and design future studies to capture meaningful impacts of SNP use.

## Materials and methods

### Research participant

The research participant was a 68-year-old male with a unilateral amputation of the right lower leg below the knee. The amputation occurred 18 years prior to study enrollment due to a non-healing wound from a motorcycle accident. In 2018, the participant received three surgically-implanted 16-channel Composite Flat Interface Nerve Electrodes (CFINEs) on his sciatic and tibial nerves (2 sciatic, 1 tibial). The electrodes were connected to percutaneous leads, which were routed through the skin in the upper leg. Electrical stimulation was sent to the nerves through the CFINEs and elicited somatosensory percepts arising from discrete locations on the missing limb that could be modulated by the applied stimulation parameters. The home study began 18 months after the participant received the implanted components of the system. Prior to entering the home study, the participant performed in-laboratory testing of his sensation and gait.

All study devices and procedures were reviewed and approved by the Cleveland Department of Veterans Affairs Medical Center Institutional Review Board, the Department of the Navy Human Research Protection Program, and the U.S. Food and Drug Administration under an Investigational Device Exemption. Study procedures and experiments were performed in accordance with the approved study protocols. Written informed consent was obtained from the participant prior to engaging in research activities.

### Sensory neuroprosthesis (SNP)

**FIGURE 1:**
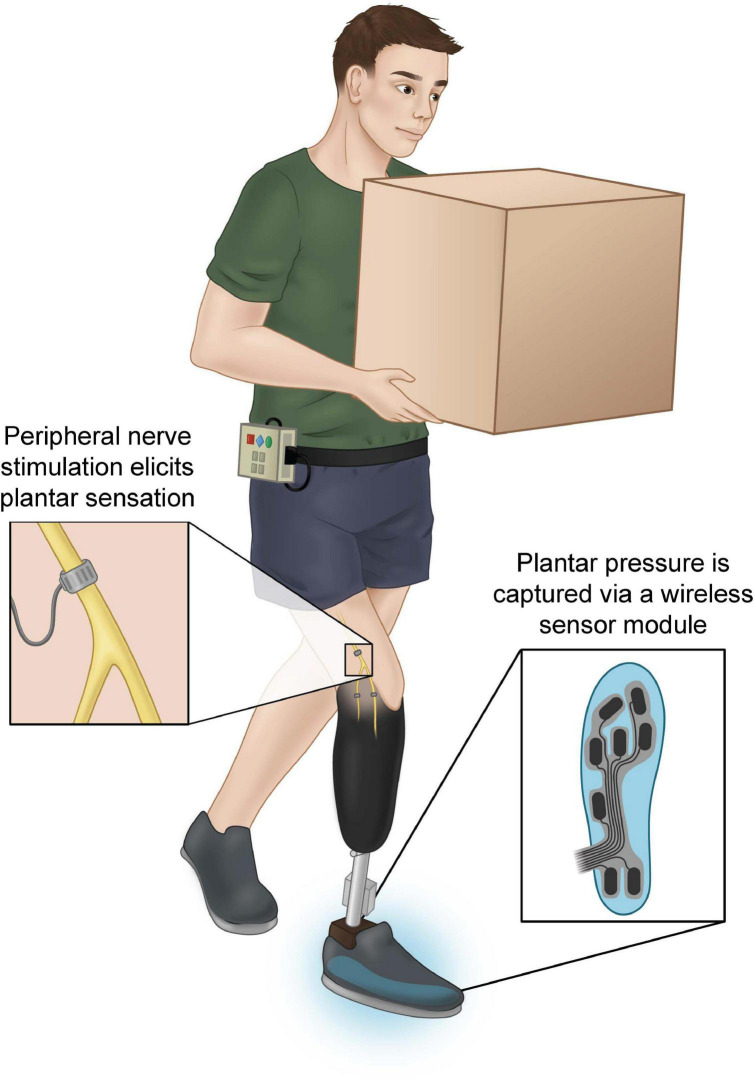
Participant with unilateral transtibial limb loss using the sensory neuroprosthesis (SNP) to perform a task in which his vision is occluded. Sensors in his prosthesis shoe **(right inset)** transduce pressures applied to the prosthetic foot during walking, standing, or other activities. Sensor signals are sent wirelessly to an external neurostimulation system worn on the belt, which then sends neural stimulation to the sciatic and tibial nerves through chronically-implanted Composite Flat Interface Nerve Electrodes (C-FINEs) **(left inset)**.

The participant wore his own prosthesis for the duration of the study with the SNP components placed onto the existing prosthesis. A sensor was attached to the sole of his prosthetic foot under his sock. The sensor measured changes in pressure in the forefoot, midfoot, and heel (Figure 1). These locations were mapped to channels on the C-FINE that elicited sensations in these areas of the missing foot. When the participant placed pressure on one of these areas of the prosthesis, stimulation was sent to the corresponding C-FINE channel, and the sensation was felt immediately on the corresponding location of the missing foot (Figure 1). The amount of pressure applied to the sensor linearly scaled the width of the stimulation pulses. A full description of the system has been reported elsewhere (Charkhkar et al., 2020; Christie et al., 2020). The investigators set the minimum and maximum allowable stimulation parameters based on in-lab-reported thresholds. These ranges were adjusted throughout the study during in-lab evaluation sessions based on participant feedback. Additionally, when desired, the participant had the ability to adjust his stimulation settings on his own at any point during the study on a per-channel basis to ensure that the sensations were supra-threshold and comfortable. The entire external assembly, including the sensorized prosthetic foot, neurostimulator, and associated cabling, constitute the lower limb SNP (Figure 1). The system had hardware and software limits to keep the applied charge within a safe range to avoid any potential for tissue damage (Shannon, 1992).

### Study design

**FIGURE 2:**
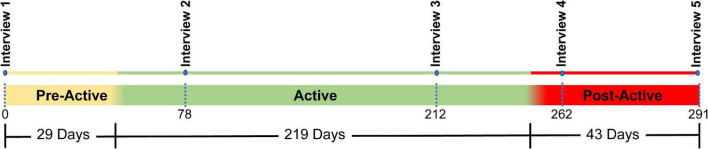
Study timeline. The total study covered a period of 291 days and was divided into three sections: Pre-active (29 days), Active (219 days), and Post-Active (43 days). Five interviews (denoted by blue dots) were conducted over the course of the study. One interview occurred at the beginning of the trial, two interviews occurred during the active phase, and two occurred during the post active phase.

The home study lasted 291 days and was comprised of three phases: Pre-Active (29 days), Active (219 days), and Post-Active (43 days) (Figure 2). During the Pre-Active and Post-Active phases, the participant wore the SNP, but it did not deliver stimulation to his nerves through the C-FINEs. The Pre-Active phase was intended to establish familiarity with the system and capture baseline experiences without sensory feedback. During the Active phase, the stimulation was enabled, and the participant was permitted to use it autonomously in his home and community. The Post-Active phase allowed the participant to reflect on his experiences with his standard prosthesis after he had experienced sensation from the SNP.

The overall study was designed as a convergent parallel mixed methods study, where quantitative and qualitative data are collected at the same time, independently analyzed, and examined to see if the results from each method can support or explain one another (Fetters et al., 2013; Creswell, 2014). This manuscript focuses on qualitative analysis of interview data, while a separate manuscript will describe the results of the quantitative functional and psychosocial measures. Semi-guided interviews were conducted at five-time points throughout the study (Figure 2). The semi-guided interviews each lasted 45–60 min and focused on the participant’s experiences with the SNP, his views on the sensations provided, and his opinions about how the SNP interacted with his prosthesis use and lifestyle. The interview questions were customized for each phase, and example questions can be found in the Supplementary Appendix.

During the first two phases, the participant was instructed on a regimen of training exercises at home to familiarize him with the sensation on each portion of his foot and to integrate the sensation with balance. The exercises consisted of: (1) weight shifting between the intact and prosthetic limb while quiet standing, (2) modified tandem stance: quiet standing with one foot staggered in front of the other with eyes closed, and (3) Stair taps: tapping the toe or heel against a step platform.

### Qualitative analysis

The five interviews were audiotaped and transcribed verbatim. The transcribed interviews were reviewed and manually compared to the recorded audio to verify accuracy (Easton et al., 2000; Mcmullin, 2021). The resulting text was then imported into NVivo 12 Plus. A qualitative analysis was conducted on the interview data to describe the experience of utilizing a sensory neuroprosthesis in the home and community using a modified grounded theory approach (Strauss and Corbin, 1998).

Three analysts (EG, JW, and MS) performed line-by-line open coding of the first two interviews to develop a preliminary codebook and definitions (Strauss and Corbin, 1998). Next, two analysts (MS and JW) implemented the code structure and definitions to analyze the remaining three interviews. The node structure and definitions were then iteratively fine-tuned through team discussion to reach a final coding structure (Table 1). MS and JW then coded all five interviews with the refined node set with consensus coding and constant comparative methods (Strauss and Corbin, 1998; Hallberg, 2006; Creswell, 2014; Cascio et al., 2019). Discrepancies in coding were discussed with EG to resolve inconsistencies. Each analyst used memos and annotations to create an audit trail (Birks and Francis, 2008). To further evaluate the relationships among the codes, axial coding was performed by breaking each code into meaningful sub-categories. The investigators did this in narrative and graphic form to understand the components of each code. Relationships between codes emerged from overlapping concepts in the sub-categories. Codes and themes were then presented to HC and later to RT for the purpose of peer debriefing (Creswell, 2014). Finally, the team performed selective coding to determine the central theme of the research, which nodes are relevant to the central theme, and which nodes were not relevant to the central theme of the research. Nodes that were not included in the model are described in the Supplementary Appendix. The team then constructed a theoretical model of the central theme.

**TABLE 1:** Coding structure and definitions for qualitative analysis.

Categories	Node	Definitions
Requirements for integration	Focus and attention	Descriptions of the amount of concentration needed to operate either the prosthesis or the sensory neuroprosthesis (SNP).
	Stimulation level	Comments about the stimulation parameters used or words/numbers describing the stimulation level, such as “high” or “low.” Comments about changes made to the stimulation parameters and associated rationale.
Sensory phenomena	Stimulated sensation–Perceived sensation	Descriptions of the quality, intensity or location of sensory percepts that were evoked by stimulation from the SNP.
	Stimulated sensation–Unpleasant sensation	Comments about undesirable, unpleasant, or ineffective sensations evoked by SNP stimulation.
	Phantom sensation–Perceived phantom	Descriptions of sensations originating from the missing limb that are not directly attributable to stimulation by the SNP.
	Phantom sensation–Unpleasant phantom	Descriptions of sensations that were unpleasant or undesirable that pertained to the missing limb and were not directly attributable to SNP stimulation, such as phantom limb telescoping.
Integration into the body	Working in the background	Descriptions of how consciously accessible the sensation was when utilizing it during daily tasks and the perceived mechanism through which the sensation impacted the participant’s prosthesis use.
	Integration process	Comments about the process through which sensation from the SNP becomes integrated into the body.
Outcomes of integration	Changes in strategies of locomotion	Descriptions of the physical and mental mechanisms that the participant would use when performing movements with the prosthesis.
	Normal state	Comments about how natural or normal the SNP stimulation and SNP utilization was to the participant, and descriptions of the conditions the participant felt were necessary for SNP-elicited sensation to be viewed as normal.
	Positive outcomes	Comments about the participant’s views regarding sensory feedback and its effects in his life, the participant’s opinions about the results of having the system, or the participant’s overall outlook on the future of the technology. Comments about expected outcomes of study participation, goals of participating in the home study, or expectations for sensory-enabled prostheses.
	Utility	Comments about the SNP system being useful, descriptions of actions or situations in which SNP-elicited sensation is useful, or examples of experiences that demonstrate the benefits of the sensory enabled system.

The research team members involved in data analysis considered how their professional backgrounds, worldviews, experiences, and assumptions might influence their analysis. The analysts identify primarily with a constructivist epistemological framing (Creswell, 2014). All five of the authors have extensive experience with neural technologies for rehabilitation. MS, HC, and RT have over 3 years of experience working with this participant in related research studies. EG has prior experience with grounded theory analysis in similar SNP studies and helped to shape the research questions. JW was brought onto the team after the data had been collected and had never met the participant. HC and RT served as external auditors and met with the team at intervals to review the results.

## Results

**FIGURE 3:**
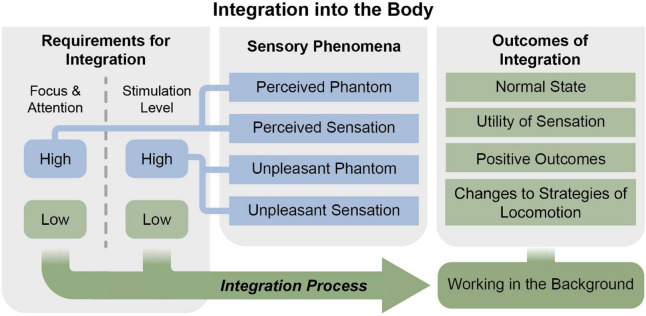
Theoretical model of the experience of integration of a sensory neuroprosthesis into the body. Gray boxes represent overarching categories and blue and green boxes depict nodes. The blue nodes include conditions and experiences which were not associated with sensorimotor integration of the sensory neuroprosthesis. The integration process (green arrow) occurred when the requirements for integration were met (green boxes, left) and resulted in outcomes of integration (right).

Through grounded theory analysis, twelve primary nodes emerged and were organized into four overarching categories (Table 1). The four overarching categories were “Integration into the body,” “Requirements for Integration,” “Sensory Phenomena,” and “Outcomes of Integration.” The central theme identified by the analysis was the “Experience of integrating a sensory neuroprosthesis into the body.” We constructed a theoretical model of the central theme, which describes the process through which a new sensory input was integrated into an SNP user’s sensorimotor processes (Figure 3). The model centered on the participant’s views and experiences regarding how the sensory neuroprosthesis modified and augmented his internal sensorimotor model, leading to several behavioral outcomes. Each over-arching category is represented as a section of the model with multiple nodes within it.

### Integration into the body

The most critical category in the theoretical model is “Integration into the body.” This category describes how the participant felt that the SNP became integrated with his natural body system in a way that he was using it without conscious thought. This major category consisted of the nodes “Integration Process” and “Working in the background,” described below.

#### Integration process

The participant described the integration of the sensation provided by the SNP into his body and motor plans as a process that occurred over time. The participant explained how the integration of the SNP into his body was “automatic” and did not require any voluntary action on his part.

*I had been using my foot to feel things and stuff and do things, without even trying to do it. It just kind of came to me normally, without any pressure, without really trying to develop it. Just having it there made its own impression, without really working at it. (Interview 4)*

The participant’s descriptions are consistent with the integration of the SNP into the sensorimotor systems of his body, which may include the body schema or other internal models. He describes how the SNP “installed itself” into the “natural reactions and systems” of his body. The participant also describes how the integration occurred when both his level of focus and the stimulation settings were low.

*It (the SNP) has worked itself into my natural system subconsciously somehow*… *I very strongly believe that I was using it (the sensation)*… *but I didn’t realize how much I was using it because it was at such a low level and my not paying attention to it*…*And I do believe that it (the SNP) has installed itself into my natural reactions and systems somehow in my subconscious. (Interview 5)*

The participant repeatedly described experiences in which he noticed changes in how he used his prosthesis when the sensation was enabled, even though he could not readily perceive the sensation. Over time, the participant reported that the integration of the SNP made it feel like the prosthesis was “a part of me.”

*Even when [I’m] not paying attention to it (the SNP), it’s working and sending the stims (sensations) in the background and stuff. And I think my system got used to it and started using it. I believe that it actually works in the background. It’s better just using it throughout the day without even paying any attention to it. It starts becoming a part of me. (Interview 4)*

#### Working in the background

The participant described how he may not always be aware of the sensation produced by the SNP stimulation when he is not concentrating on it, but he believed that this imperceptible sensory input was still “working in the background” to impact his prosthesis use. He felt this enabled the sensory neuroprosthesis to integrate into his body and sensorimotor processes.

*If I am concentrating on it (the sensation), I am confident that it is there and that I am using it. When I am not concentrating on it, I can only assume that it is working in the background and that I am using it. That is where I believe the sensation works without me concentrating on it. (Interview 3)*

Working in the background describes the situation in which stimulation was applied to the nerves *via* the SNP but without the participant concentrating on or paying attention to it in any way, resulting in him being unaware of the sensory input.

*It is different now that I am using very little stims (low stimulation levels), instead of the high stims (high stimulation levels) because once they (the sensations) turn on, I have to concentrate very, very, very hard to feel them on the toe and the midfoot. But I believe they still work in the background even though I am not paying attention to them. (Interview 3)*

### Requirements for integration

The participant described two criteria upon which integration depended: (1) the amount of focus or attention he exerted on the prosthesis or neuroprosthesis and (2) the level of stimulation provided through his neuroprosthesis. The participant explained how his experiences of the sensory neuroprosthesis and the ability to integrate the provided sensation into his motor plans differed when the focus was “high” vs. “low” and when the stimulation level was “high” vs. “low” (Figure 3). Focus and stimulation level also interacted, such that experiences with high focus and high stimulation level differed from experiences with high focus and low stimulation level.

#### Focus and attention

Focus describes the mental energy or effort the participant applied to use the SNP or to feel the sensation provided by the neuroprosthesis. For example, the participant reported that in many cases, he needed to concentrate on the SNP to clearly perceive the sensation it produces.

*I got to think about it [the sensation] and pay attention to it to feel it, but it is there. (Interview 1)*

He also reported difficulty perceiving the sensation provided by the neuroprosthesis when stimuli in his environment disrupted his concentration.

*My concentration level has to be so high to recognize it (the sensation). The other inputs of walking, people being around me moving, it all distracts me from being able to get into [the] zone, my zone I call it*…. *At home when I have the T.V. off, nobody talking, nobody bothering me, and I’m really into my zone, I can do a lot more with it (the sensation). (Interview 2)*

The participant described how he typically did not apply a lot of attention to his prosthetic foot in day-to-day life, and similarly, he did not focus on the sensation while walking around and doing other tasks. Instead, he reported needing to stop activities and concentrate on the sensation to perceive it.

*As I start moving and working around–doing other things–my concentration goes on to what I am doing, and I don’t really pay attention to the foot, except once in a while when I stop to concentrate and check on it. (Interview 3)*

#### Stimulation level

**FIGURE 4:**
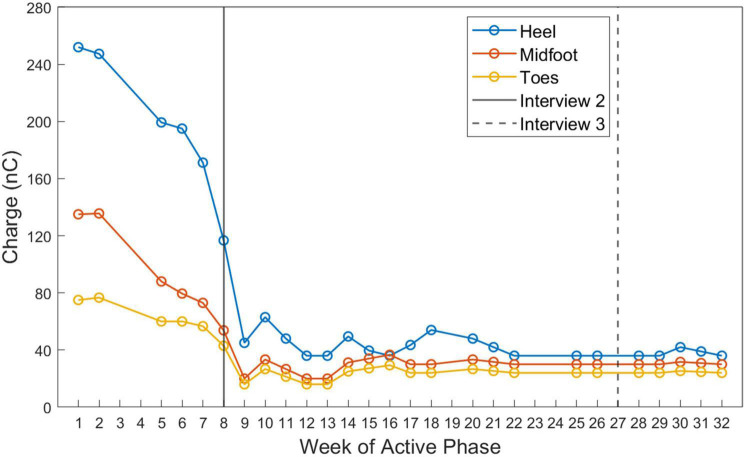
Stimulation settings selected by the participant during the Active Phase of the study. The charge delivered during the cathodal phase of each stimulation pulse is displayed for each week of the Active Phase. Charge was calculated by multiplying the pulse width and pulse amplitude parameters selected by the participant on a given day. Color indicates the stimulation contact associated with the sensors in the heel (blue), midfoot (red), and toe (yellow) of the prosthesis. The timing of interviews 2 and 3 relative to the Active Phase are displayed as vertical lines.

The SNP minimum and maximum stimulation parameters for the toes, midfoot, and heel were set by the research team to levels that the participant had previously deemed to be comfortable and that he had previously used for in-lab testing (Charkhkar et al., 2020; Christie et al., 2020). The participant was also given the ability to adjust the stimulation for each of the three areas provided *via* the SNP to account for any day-to-day fluctuations in his sensory detection thresholds so that he would be able to continue feeling the sensation throughout the study. The stimulation settings for all three sensory percepts (toes, midfoot, heel) decreased in charge per pulse throughout the Active Phase of the study period (Figure 4).

The participant found that the stimulation levels he typically used for in-lab testing were too strong for prolonged use at home. Early in the study, when he set his stimulation levels to be “as high as I can and tolerate them,” he reported that the sensations he experienced were very strong. He explains that he selected these higher stimulation levels because he thought that he “needed to feel [the stimulation] as best as possible.”

Later in the study, the participant decided to lower the stimulation settings, which changed his sensory experience to that of a “light tickle” or a “tingle tickle almost.” The participant preferred the low stimulation settings, saying that it was “working maybe better” than the high settings.

When the stimulation levels were low, the participant only perceived sensation when his concentration was high. If the participant did not concentrate on the SNP stimulation, he was generally unaware of the sensory input.

*If I really want to feel the stims at that low level, I kind of stop and concentrate and feel for them*… *have to concentrate on that a lot more because it [the sensation] is so much lighter. (Interview 4)*

### Outcomes of integration

The outcomes of sensorimotor integration of the SNP included changes to strategies of locomotion, returning to a more normal state, increased utility of the sensation, and overall positive outcomes (Figure 3).

#### Changes to strategies of locomotion

The participant changed the way that he ambulated and used his prosthesis when sensation was enabled in the SNP. He described how he previously would “stomp around” or “slam” his prosthetic heel down into the ground so that he could feel the reverberations up into his socket. However, with sensation enabled, he did not need to utilize this walking strategy and could instead put much less weight into his prosthetic leg.

*So, I feel it (weight, pressure) up through my residual limb and my leg and into my body in a different way than when I don’t have the stimulation on. And I have had these experiences where I’m not really putting my weight on my foot, I’m standing there with only partial weight on it on the ground. It’ll be on the ground, but I don’t have enough weight on it to really feel it up through my residual limb and stuff. And I’m depending on that stim (stimulated sensation) to come. (Interview 5)*

The participant developed a new strategy of using his prosthesis during the home study. When his vision was occluded or he was walking backward on steps or uneven terrain, he would use the toe of his prosthetic foot to tap the ground and feel the sensation provided by the SNP to identify where it was safe to step with his prosthesis.

*I was taking the air conditioners out of the windows in my house and I had them pushed up to my chest and was walking across the yard in the grass and I couldn’t see the ground. And I found myself doing the same thing there, tapping the ground with my foot, and not really taking a step because I had stopped and was searching for the ground with my right [prosthetic] foot. (Interview 5)*

Changes to utilization of his prosthesis occurred without voluntary effort or training. The participant described how his method of identifying uneven areas of the ground by tapping the ground with his prosthetic toe was not a purposefully designed strategy but rather happened “automatically.”

*I wasn’t aware that I was doing it, or even trying to do it. I just automatically was standing there, automatically tapping my toe trying to find the stim (stimulated sensation) and I didn’t even realize it until a little bit after I’d done it. Wow, this is what was happening! (Interview 3)*

#### Normal state

Integration of the SNP into the participant’s body enabled him to return to what he viewed as a more normal state. The participant commented that low stimulation levels were critical for the sensations to be perceived as normal.

*I think I am closer to being in a normal state with them (the sensations) turned down low like this*…*I don’t pay attention to them, but I think the low stims that I am using now are kind of becoming that for me. Becoming normal rather than a distraction. (Interview 3)*

He described how the sensation provided by the SNP contributed to his “normal operating system” to help him with his daily activities. When he didn’t have sensation, he had to rely on other inputs, such as vision or pressure in the socket, to effectively ambulate.

*It (the sensation) seems to become a normal part of my system rather than trying to, having to look for it, and make sure it’s there and try to use it. Somehow, I think I just automatically do it*…. *I’m talking just like a normal operating system*… *It (the sensation) is working in the background back there. And when it’s not there working, I have to rely like on a different type of input. (Interview 4)*

The automaticity with which he was able to use the sensations provided by the SNP was a key factor in his belief that it helped him to return to a normal state. Demonstrating how normal the system had become for him, he compared the way that he used the SNP to the way that he used his intact, left leg.

*But to me*… *that seems important that you can actually get the benefit and use the system when it’s set up and adjusted and the stims (stimulated sensations) are at the proper levels without really even noticing. It just becomes automatic like my left leg is in a way. (Interview 4)*

#### Utility of sensation

The participant found the sensation provided by the SNP to be useful in helping him to walk and perform locomotion tasks. He reported that the sensation received from the SNP, especially from the toe of the prosthesis, was useful for assessing the ground for “irregularities” that could impact his ability to safely ambulate.

*I do believe that I have been using probably the toe on my foot more than anything to find depth and irregularities in the ground. I can tell that the toe is the most useful sensation, like when I am looking for a step. Or when I am looking at uneven ground when I am pushing the lawn mower*… *I am using it with my toe to find an uneven spot on the ground. (Interview 3)*

The participant felt that his ability to use the sensation automatically without having to concentrate was especially important for the usefulness of the SNP.

*I believe it (the sensation) might be getting more and more useful even when I’m not paying attention to it. That’s what seems to be the important thing to me is that it (the sensation) is engraining itself into my subconscious when I’m not paying attention to it. (Interview 3)*

At the end of the study, the participant summarized some of the key ways in which the sensation provided by the SNP was useful to him. He also reflected on his experiences using the prosthesis for the final portion of the study without sensation enabled and how he realized that he had “started to depend on” the sensation provided by the SNP.

*The value to it (the SNP) for me is being able to feel the ground. I don’t know if it makes me walk better, but it makes me aware of where my foot is at when I need to know. That is a very, very positive thing and I like it. Especially since I missed the sensation a few times and I realize how much I was probably using it and depending on it when I had it on. (Interview 5)*

#### Positive outcomes

Overall, the participant had positive experiences with the SNP, especially after he identified optimal stimulation settings, he accumulated several months of experience using the SNP, and the sensorimotor integration process had occurred. When asked what he thought about the sensory feedback provided by the SNP, he said:

*Well, I think it’s a pretty amazing thing*… *and it’s what it’s done in my subconscious that is more amazing than when I’m trying to pay attention to it all the times*… *It feels good just to have it there I guess, and know it’s working, and I am using it*…*I think it exceeded my expectations. (Interview 3)*

The participant viewed the integration of the SNP into his body as a key accomplishment, because it enhanced the naturalness and usability of the prosthesis.

*That’s what I think is the most valuable part of it [the SNP] is that it becomes this natural, useful thing. (Interview 3)*

The participant described how without the SNP, he felt less whole or like “some part of me is missing.”

*It feels like there’s a little bit of something missing. I don’t know how to describe it. It’s not that I can’t function without the stims (stimulated sensations), but I just feel like there’s an empty spot in my system somehow*… *When it’s not there I feel just a little bit like some part of me is missing somehow. (Interview 4)*

The participant stated that he “definitely prefers having the sensation” from his prosthetic foot and stated that he would participate in another sensory restoration study if given the opportunity.

### Sensory phenomena

The participant described several different types of sensory phenomena throughout the study. We broadly characterized these as sensations of his phantom limb and sensations elicited by the neural stimulation (Figure 3). Interestingly, the integration process through which the SNP became a part of the participant’s body appeared to be impeded by readily perceivable sensation either in the phantom or due to stimulation.

The sensation of his phantom limb was sometimes neutral (perceived phantom) and sometimes unpleasant (unpleasant phantom) and often interacted with his experiences with the sensory neuroprosthesis. Many of the sensations he experienced originated directly from the neurostimulation applied to his nerves *via* the SNP. We divided these stimulation-elicited sensory experiences into two categories: (1) perceived sensation (neutral or positive valence), and (2) unpleasant sensation (negative valence).

#### Perceived phantom

Prior to and during the study, the participant had an experience of a phantom limb. He described his phantom sensation as a light tingling sensation, that was co-located with the prosthetic foot (“it is right where my prosthesis is”). When he was asked to describe the quality and intensity of his phantom sensations he replied:

*When I think about it, [the sensation on the phantom] is just a little tingling here and there, and it kind of moves around and varies a little bit. It is very light. (Interview 1)*

The participant’s ability to move and perceive his missing foot, ankle and calf varied by body region. He described that he could generate a sensation of movement within the phantom toes but does not perceive a phantom calf and cannot move his phantom ankle.

*I have a phantom ankle that’s locked up tight. I don’t have much [sensation] of the phantom leg above my ankle. The leg is not really there, it is all from about the ankle down. I can curl my toes. (Interview 1)*

The phantom sensation was perceptible and comfortable when the stimulation levels were low, and the participant concentrated on his phantom limb.

*It (the phantom) doesn’t come forward and make a presence unless I think about it. It is something I have to think about a little bit in order to even tell it is there. (Interview 1)*

#### Unpleasant phantom

The SNP affected the participant’s perceptions of his phantom limb. When the stimulation delivered by the SNP was high, especially for prolonged periods such as when higher pressures were continuously applied to the sensors by standing still on the prosthesis, the phantom foot became uncomfortable.

*And if I stand flat footed on my prosthesis, I can feel all of them (the sensations from the SNP) at once. If I stay there [standing still] for long it just takes my muscles in my stump and my ankle on my phantom foot and just seems to tighten and lock everything up*… *a crampy kind of feeling. (Interview 2)*

Rather than only modifying the phantom when the stimulation was on, the uncomfortable phantom experiences persisted beyond the presentation of stimulation.

*When they (the sensations) are at the high level*… *they did not bother me throughout the day, but they left a lasting effect when I took it (the prosthesis) off. (Interview 3)*

These unpleasant phantom experiences included an experience of phantom telescoping, in which the phantom foot would retract into the residual limb. This was a very disturbing experience for the participant and gave him “nightmares.” The participant described how the high stimulation levels gave him unpleasant phantom experiences that persisted throughout the night and disrupted his sleep. However, this did not occur when the stimulation level was low.

*You know when I was started out, I started out running [the stimulation] as high as I could, trying to see how well-defined I could feel [the sensations], and it was giving me all those troubles at night and the stims (phantom sensations) were staying on all night long. Keeping me up and giving me problems*… *when I felt like my foot moved up to the bottom of my stump and things like that. Since I have been running these (the stimulation) down low, I have not had any of that. (Interview 3)*

#### Stimulated sensation–Perceived sensation

As expected from prior psychometric studies of sensory stimulation (Graczyk et al., 2016, 2022; Charkhkar et al., 2018), the sensation modality and intensity depended strongly on the stimulation levels set by the participant. When the stimulation levels were low, the participant described the stimulation-elicited sensations as “tingly” or “a very light tingling.” When the stimulation levels were high, the participant explained how the sensation modality changed:

*When I had the sensations turned up high it was a stronger tingling, almost a prickling feeling. (Interview 3)*

The research team mapped each sensor in the shoe worn on the prosthetic foot to a corresponding stimulation channel that evoked a matching perceived location. The participant was able to detect changes in the position of the perceived sensation as he applied pressure (or “rocked”) to different parts of his prosthetic foot.

*But when I rock to the outside of my [prosthetic] foot, it feels like I have pressure or a tingling sensation right down this side of my foot. It’s not in my stump at all. It seems to be in my foot and when I rock toward the inside, it seems to move across to my inside in the whole length of my foot. (Interview 2)*

#### Stimulated sensation–Unpleasant sensation

Although the stimulation parameters were initially set at comfortable and safe (Shannon, 1992) levels previously selected for in-lab testing, the participant voluntarily turned down the stimulation levels after the first several weeks of the study, after finding them to be too high for regular at-home use. The participant reported that the initial or “high” stimulation levels were associated with uncomfortable or unpleasant sensations.

*That stimulation level, when it was high it, like I said it took my light tingling sensations that I work with comfortably and turned them into a harshness, like a buzz. (Interview 2)*

The participant also said that the high stimulation levels caused diffuse sensations across his entire foot that blurred his ability to discriminate individual areas of his foot. This may have reduced the informativeness of the sensations produced by the SNP when stimulation levels were high.

*It goes past tingling into like a harsh buzz. And then it encompasses my whole stump and foot*…. *They (the sensations) were getting so excited and jammed up all into one big ball*…*Jammed up just means it feels like all the stims (the sensations) are on at the same time and it’s working my whole stump and foot instead of individual areas. (Interview 2)*

The high stimulation levels also increased the intensity of the sensation in his residual limb, which made it difficult to perceive the sensation from the SNP.

*And that to me is one of my biggest problems with it, it doesn’t take long before I get so– the stump gets so revved up or stimmed up or whatever you want to call it that I can’t feel through it. (Interview 2)*

## Discussion

In this study, a unilateral trans-tibial amputee used a SNP autonomously in his home and community for 31 weeks. To our knowledge, this is the first documented home study of a lower limb SNP with implanted neural interfaces. We performed a qualitative analysis to understand the participant’s novel multifaceted experience with the SNP. The semi-structured interview approach allowed the participant to guide the discussion to topics he found salient and allowed us to understand the user’s experience holistically. Our analysis uncovered the relationship between stimulation levels, attention, and sensorimotor integration of the SNP, and how integration of the SNP into the body promoted sensation utility, increased prosthesis naturalness, and improved locomotion strategies. In the context of translation of this technology for rehabilitative and clinical applications, the user’s feedback on the benefits and limitations of the intervention will lead to iterative refinement of the technology to improve user outcomes.

### Implications for sensorimotor learning

Sensorimotor integration of the SNP resulted in the participant changing how he used his prosthesis during gait. One notable example of these changes was the use of a toe-tapping strategy to detect differences in the ground to aid his passage over uneven terrain, especially when his vision of the ground was occluded (see Figure 1). After integration, the participant frequently reported that his utilization of the sensation from the SNP and his performance of sensory-guided tasks, such as toe-tapping, occurred “automatically.” Automaticity is a hallmark of successful skill acquisition and occurs when the performance of the task is minimally affected by other simultaneous tasks (Poldrack et al., 2005). In many models of motor learning, task acquisition is divided into three stages: the cognitive, associative and autonomous stages (Fitts and Posner, 1967). During the first stage of task acquisition, intentional focused motions are required (Luft and Buitrago, 2005). In the second stage, these motions become stored as procedural knowledge, and through repeated practice in the second and third stages the amount of focus required to perform the task diminishes until automaticity is achieved (Anderson et al., 1997; Wickens, 2014; Tenison and Anderson, 2016). Thus, the automatic toe tapping and other walking strategies described by the study participant suggest that he had successfully undergone sensorimotor learning with his new prosthesis.

Though the participant had previously utilized the SNP in a laboratory context and performed tasks including motor imagery, postural stability (Shell et al., 2021), treadmill walking, and walking overground (Christie et al., 2020), the emergence of automatic strategies to specifically gather sensory information, such as toe tapping and rocking on the prosthesis, only occurred after several months of daily use of the SNP. The implication is that in lab training alone may not be sufficient for motor learning to reach the level of automaticity, and thus daily at-home training may be required. In addition, this training process may need to occur over the course of several months for new locomotive strategies to become automatic.

While the participant reported that his toe-tapping strategy emerged “automatically” without intentional planning on his behalf, the strategy did resemble a prosthesis exercise he was trained to do by study staff to familiarize himself with the SNP-elicited sensation early on in the study. It is unclear if experience with this training paradigm was necessary for the participant to be able to implement the strategy in unstructured settings, or if he would have spontaneously developed the strategy on his own. Regardless, targeted laboratory training coupled with home training is likely to improve the probability of sensorimotor learning and successful SNP outcomes. Most of the targeted exercises employed in this study were simple movements, such as shifting weight onto different portions of the foot, without specific instructions to implement them during mobility related activities of daily living. The training exercises for SNP users could be expanded to include specific task-related applications to gain optimal sensory information. For example, participants could be trained to perform toe tapping to feel irregularities in the ground, based on the findings from this study. This participant’s experience with utilizing specific strategies to collect sensory information with the SNP to aid his locomotion could help inform future research into effective sensory training regimes for lower limb SNPs.

Prior studies on other sensory neuroprosthetic devices have employed a similar approach developing training regimes around specific actions that will aid in collecting optimally useful sensory information. For example, individuals with congenital blindness using visual prostheses coupled with a head-mounted camera were expressly taught how their head movement would impact the collection of data by the camera and how this motor task is mapped to the generation of important visual concepts such as depth perception (Guarniero, 1974). Similar motor training occurs in the use of the Argus II retinal implant (Markowitz et al., 2018) and a visual-to-tactile sensory substitution system for the visually impaired (Chebat et al., 2011). Future SNP studies could provide participants with explicit training in how different signals from the prosthesis relate to terrain characteristics. As shown in prior research of sensorimotor learning with other sensory augmentation devices, this targeted training is expected to improve the users’ proficiency with the sensory information and should allow users to more skillfully perform tasks with the SNP. Improving sensorimotor integration of SNP-provided sensation may further enhance the functionality of lower limb prostheses during activities of daily living in a variety of environments.

### Implications for perceptual learning

Perceptual learning is an experience-dependent process through which one’s ability to extract sensory information from stimuli and interpret their meaning improves due to practice (Gibson, 1963; Sathian, 1998; Seitz, 2017). This process allows for better detection and interpretation of weak or ambiguous stimuli (Gold and Watanabe, 2010). The participant reported that stimulation levels that were previously deemed useful for in-laboratory testing became uninformative and uncomfortable after several weeks of use. After a few weeks of the home study, the participant described these initial stimulation levels as “high” and reported detrimental effects to his phantom experience. He subsequently chose to lower the stimulation levels to improve his sensory experience (Figure 4). Interestingly, these “low” stimulation levels were not deemed useful or readily perceivable during the baseline testing at the start of the study. This change in the perceived intensity and utility of the sensation between the baseline testing and after several weeks of home use suggests that perceptual learning occurred, in which the user became better at identifying the provided sensation and discriminating changes between sensory percepts provided at these lower levels.

Perceptual learning has been documented in other sensory neuroprostheses. For example, cochlear prosthesis users’ abilities to identify vowels, consonants, and sentences have been shown to improve over time (Fu et al., 2002). Cochlear prosthesis users achieved the best learning results when the incoming stimulation was aligned optimally with the tonotopy of the cochlea, leading to faster improvements in sound recognition and higher peak performance (Fu and Galvin, 2007). In the current study, SNP stimulation channels were selected such that the sensations elicited by stimulation were somatotopically aligned with the sensors in the prosthesis shoe. As described above, this somatotopic alignment was sufficient to enable some degree of perceptual learning. The size of the evoked percepts may also have played a role, since perceptual learning was not observed at the higher stimulation levels, which tended to produce large, diffuse percepts. These large percepts overlapped across multiple sensors, and thus were less informative about the location of contact between the prosthesis and the ground. It is also possible that perceptual learning could have been further enhanced if other aspects of the evoked sensations, such as their perceived quality, intensity, and naturalness, were also matched more closely with the transduced sensor information. Improved perceptual learning of the SNP could potentially lead to better discrimination between forces applied to the prosthesis or faster detection of weak stimuli applied to the prosthesis. Future research should investigate stimulation paradigms that improve the quality and perceived naturalness of the elicited sensation to promote perceptual learning and examine how training tasks can be designed to help SNP users learn to interpret the provided sensory information.

### Implications for embodiment

Embodiment is a multifaceted concept that describes how an individual perceives their body and the actions they make with it (Schettler et al., 2019). Studies of traditional prostheses (Murray, 2004), dexterous upper limb prostheses (Luchetti et al., 2015), and Osseointegrated prostheses (Lundberg et al., 2011) have also demonstrated the importance of embodiment for prosthesis users. Furthermore, the relationship between electrically elicited sensory feedback and prosthesis embodiment has been investigated in prior studies (Mulvey et al., 2012; Collins et al., 2017).

One critical component of embodiment is the body schema, a preconscious internal model of the body, available motor plans, and predictions of the sensory consequences of actions (Maravita and Iriki, 2004). Our model shows how integration of the SNP-provided sensation into the user’s sensorimotor processes, which likely includes integration into the user’s body schema, led to improvements in functional outcomes. The participant repeatedly describes how the sensation “installed itself” into his “subconscious systems” and that the sensation was “working in the background.” These statements likely reflect the participant’s attempts to describe his subjective experience of his body schema, which largely operates outside of conscious awareness. That the participant could articulate an experience of his body schema at all demonstrates how pervasive and meaningful these experiences were to him.

In addition, the semi-guided interviews did not include any questions specifically addressing embodiment of his prosthesis or components of embodiment, such as agency and ownership (Zbinden et al., 2022). Thus, our findings related to embodiment are limited to what the participant self-reported. Our study also did not assess prosthesis embodiment or the participant’s experience of his body schema consistently across the study. Thus, we cannot address the specific time course over which the experiences of the body schema changed or over which the integration process occurred. Future qualitative studies of SNP use should include interview questions focused on understanding all of the components of embodiment and changes in embodiment experiences across the course of the study.

### Comparison to prior take home studies

This study expands on a previous series of take-home studies of an upper limb SNP, which also utilized qualitative analysis with grounded theory approach to develop theoretical models explaining the benefits of sensation on the prosthesis experience (Cuberovic et al., 2019; Graczyk et al., 2019). These studies produced models which explored how the naturalness and usefulness of the restored sensation contributed to the user’s confidence in their abilities, prosthesis embodiment, and outcome acceptance, and how sensation reduced the focus and attention required while using the prosthesis (Graczyk et al., 2019). The model in this study can be directly compared to these prior models to provide a basis for understanding the different needs and experiences of upper vs. lower limb prosthesis users related to touch feedback.

Consistent with the previously reported models, the model in the present study includes nodes related to focus and how sensation seems to interact with and alter the attention required to use the prosthesis successfully. In the upper limb studies, researchers observed that the participant became increasingly comfortable with the sensation and utilized it more efficiently (Cuberovic et al., 2019). This experience is analogous to the changes in locomotion strategies in the present study, in which a proper level of stimulation and attention interacted to let the user effectively utilize the sensation. Additionally, both upper and lower limb models included ideas related to embodiment. In the prior upper limb studies, the participants’ embodiment experiences were predominantly related to the concept of ownership, wherein they reported that the prosthesis was “my hand” or that it was part of them. In the present study, the participant did not report many experiences relating to prosthesis ownership, but the integration of the sensory feedback into the body likely reflects the participant’s experiences of the preconscious body schema.

One key difference between this study and prior investigations of upper limb SNPs during home use is the involvement of automaticity and subconscious sensorimotor processes. The integration of the SNP into subconscious and preconscious processes and the automaticity of prosthesis use were central elements of the participant’s experiences in this study that were not discussed by prior upper limb prosthesis users (Cuberovic et al., 2019; Graczyk et al., 2019). This may be due to the fact that the use of upper limbs is often more intentional than lower limbs, as they are used to actively interact with the world for goal-directed tasks, such as grasping a cup or performing a handshake, whereas lower limbs are used for repetitive, semi-automatic, often rhythmic behaviors, such as walking. Thus, the degree of automaticity of prosthesis use may have been less relevant to the upper limb users in prior studies.

### Implications for the phantom experience

Secondary outcomes of our model show that long-term SNP use can shape a user’s phantom experience. Previous work has suggested that peripheral nerve stimulation may be a potential treatment for phantom pain (Dietrich et al., 2012; Tan et al., 2014; Johnson et al., 2015; Petrini et al., 2019). Furthermore, past home studies have shown that extended SNP use reduced limb-telescoping, or the unnatural positioning of the phantom limb (Graczyk et al., 2018; Cuberovic et al., 2019). Generally, phantom experiences are believed to depend on somatosensory cortex reorganization (Grüsser et al., 2001; Flor et al., 2006), which results from the absence of or irregularities in sensory information due to the limb loss. Our model outlines how the pleasantness of the phantom experience can be modulated by application of sensory stimulation by the SNP. Rather than improving the phantom experience, as prior studies of phantom pain have shown, our findings show that misaligned sensation levels can potentially lead to undesirable phantom experiences. During the early stage of the study, the participant experienced periods in which the phantom was uncomfortable or telescoped, even during times he was not using the SNP. This appeared to be mainly due to his preference for “high” stimulation levels during this period. Although acute, in-lab use of the same self-selected higher stimulation levels was not uncomfortable and did not result in changes in phantom perception, the prolonged use at these settings led to the undesirable phantom experiences. Upon finding lower levels of stimulation which were more pleasant and facilitated integration, these negative experiences dissipated, and the phantom sensation returned to being regarded neutrally. These findings demonstrate that persistent input from the periphery affects the perception of the phantom and may not occur instantaneously.

### Limitations

The present investigation was a qualitative case study with a single participant using an SNP in his home and community for 291 days. While qualitative methodology allows for an in-depth understanding of experiences, it is not intended to generate results that are generalizable to a broad population. Additional data collection, including quantitative and qualitative approaches, in a larger cohort of SNP users, is necessary to gain generalizable results. Such efforts would enable a refinement of the theoretical model generated in this study so that it can be appropriately applied in other research or clinical settings. Future studies of SNP use would benefit from more frequent interviews throughout the study period to increase our understanding of the time course of sensorimotor integration and learning processes. In addition, future studies should also include quantitative perceptual and motor learning measures to triangulate findings from the qualitative analysis. While this study provided evidence that the participant’s embodiment of the device changed over time, we did not collect quantitative data to elucidate the extent of this change or what specific embodiment domains were affected. Study interpretation is also limited by the implementation strategy for the SNP, which allowed the participant to freely alter the maximum stimulation parameters to levels that did not cause pain or tissue damage but might exceed those providing useful sensory information. Although the participant eventually found “lower” stimulation settings that were useful and promoted positive outcomes, we do not know if other stimulation settings or calibration procedures may have expedited the integration of the sensation or led to better outcomes than those reported here. More work is required to better understand the relationship between stimulation settings, the ability of SNP recipients to interact with and manipulate them in home and community settings, and their effects on learning and sensorimotor integration. Better participant education on stimulation settings that encourages the identification and use of an optimal system configuration may also improve outcomes.

## Conclusion

Neural-interfacing SNPs are a new intervention with the potential to restore useful somatosensory feedback to lower limb prosthesis users. In this study, we used qualitative methods to examine one user’s self-reported experience with a lower limb SNP at home for nearly 8 months. The analysis revealed that the SNP-generated sensations were integrated into the user’s sensorimotor processes and prosthesis-related motor strategies. Our analysis demonstrated that the application of stimulation-elicited sensation related to foot-floor interactions allowed the participant to devise and utilize new gait strategies, such as using lighter pressures in the prosthesis socket, which potentially demonstrates a return to symmetry in gait. This is the first report of a long-term home study of a lower limb SNP and the first user-derived model of lower limb SNP usability and functionality. The model generated from our analysis can inform prosthesis developers, researchers, and rehabilitation clinicians about the needs of lower limb SNP users and may lead to improvements in neuroprosthetic technology development and application.

Results suggest that the integration of the SNP-generated sensations into a user’s motor plan is a process that occurs over time, which highlights the need for longer-term studies to accurately evaluate new prosthetic technologies. In addition, integration may only be possible when certain requirements are met, such as a balance of cognitive effort and stimulation levels. The integration process led to an increase in the intuitiveness of using the prosthesis, making it feel “automatic, like my [intact] leg.” Automaticity is highly important in walking, with specialized pattern generators in the CNS devoted to generating automaticity in typical walking behaviors (Clark, 2015). The participant’s experiences of automaticity suggest that sensorimotor learning occurred throughout the study. Further, the notable changes in the perceived sensation throughout the study suggest that perceptual learning may have occurred. These learning processes could be enhanced in future SNP implementations through targeted training regimes, advanced stimulation paradigms, and improvements in device design.

Importantly, the participant believed that the sensation provided by the SNP was valuable and improved his prosthesis utilization. At the end of the study, he commented that he believed that future clinical SNP systems would benefit him and others like him, stating “I really believe that [the SNP system] is going to help other people someday.”

## Data availability statement

The original contributions presented in this study are included in the article/Supplementary material, further inquiries can be directed to the corresponding author.

## Ethics statement

The studies involving human participants were reviewed and approved by the Cleveland Department of Veterans Affairs Medical Center Institutional Review Board, the Department of the Navy Human Research Protection Program, and the U.S. Food and Drug Administration under an Investigational Device Exemption. The patients/participants provided their written informed consent to participate in this study. Written informed consent was obtained from the individual(s) for the publication of any potentially identifiable images or data included in this article.

## Author contributions

EG and MS designed the study, collected the data, developed the qualitative analysis strategy, analyzed and interpreted the data, constructed the model, and wrote the manuscript. JW analyzed and interpreted the data, constructed the model, and wrote the manuscript. RT contributed to the study design and revised the manuscript. HC contributed to the study design and interpretation of the data and revised the manuscript. All authors contributed to the article and approved the submitted version.
