# Supplementary material for: The experience of sensorimotor integration of a lower limb sensory neuroprosthesis: A qualitative case study
Source: Front Hum Neurosci. 2023 Jan 11;16:1074033. doi: 10.3389/fnhum.2022.1074033 (PMC9874950; doi:10.3389/fnhum.2022.1074033)
Supplement: Supplementary file 1 [file Data_Sheet_1.pdf]

Appendix 1: Node Reports

This section contains the node reports for all nodes not included in the theoretical model of sensorimotor integration of the lower limb sensory neuroprosthesis. There were six nodes that emerged through the qualitative analysis and were included in the final coding structure but were not essential to the central focus of the model and were thus not described in the main manuscript.

Doubt

While adjusting to the Sensory Neuroprosthesis (SNP) the participant seemed to be unsure about its capabilities and, at times, questioned the sensation he was experiencing. Quotations describing these uncertainties were coded in the "Doubt" node.

In the early interviews, the participant expressed uncertainty about whether the sensations he experienced resulted from SNP stimulation or from his imagination. He described this uncertainty with words like "imagined" and "dreaming" when referring to the sensation he experienced. These quotes were most prominent in early interviews when the sensation was new to the participant.

> *And I don't know if I'm dreaming that or whether [the sensation is] actually happening. (Interview 2)*

In addition to whether the stimulation was real or imagined, the participant questioned if the SNP could produce the sensation he experienced. This doubt was typically in the context of whether specific foot movements would activate the sensors in his prosthesis shoe to produce a given sensation. For example, he notes that performing a "rocking" movement would cause the sensation to move from the back of the foot to the front of the foot, and then to the inner aspect of the foot. The participant expressed the following uncertainty during one of the early interviews:

> *When I rock toward the inside [of my foot] it (the sensation) seems to move across to the inside of the whole length of my foot. …I don't know if that pressure pack (sensors) can send out [that sensation]. You know, I've got sensors on both sides [of the foot] down there. But I don't know if [the SNP] has the ability to send me that effect or not. But I do believe I am feeling it. (Interview 2)*

The participant's unwillingness to recalibrate the system in the early phases of the study was also an expression of his uncertainty with the system's operation and capabilities. He worried that recalibrating the system would cause him to lose the sensations he had become accustomed to and might require him to relearn a new set of sensations.

> *My problem with recalibrating - I don't want the levels changing that I work with all the time and have to find new ones. (Interview 3)*

Though the underlying cause of the participant's doubt was unclear, the participant expressed concern that he may not get the full effect of the system if he was not able to use the sensation correctly.

> *But it bothers me a lot with the thought that I'm not getting the full effect of the stims (stimulated sensations) and being able to work with them. (Interview 2)*

Inconvenient and Bulky

The "Inconvenient and Bulky" node contains references to components or properties of the SNP system that made the device difficult to use, uncomfortable, or hard to trust. The participant reported that the SNP was cumbersome and would often impede his ability to move around. This would occur when the SNP limited his freedom of movement or when certain movements required that he reposition system components. He also worried about damaging system components inadvertently.

> *It's a been a pretty good experience most of the time. The box on the belt [the stimulator] is a bit awkward and a pain in the butt at times, trying to keep track of it and make sure it is not getting damaged. Trying to work around it when I work out or sit up or sit down. I have to move it from the front around and slide it around to my side. When I bend over, when I am swinging my arms and doing stuff then I have to move it back around to the front and stuff. (Interview 3)*

Another source of inconvenience was the tendency for the cabling of the device to become disconnected or for buttons to be pressed, thereby changing the SNP settings, without the participant's knowledge. This accidental disconnection made it difficult to trust how long or efficiently the box delivered stimulation**.**

> *That's what bothers me when I have had it on for hours and all of the sudden, I find it turned off or unplugged, or something… And it kind of seems futile to wear it all that time and not be productive with it somehow. (Interview 3)*

When he was asked what changes that he'd suggest making to the SNP to be more user-friendly he suggested miniaturization of the device to help protect it from damage or from being accidentally deactivated or unplugged.

> *Getting the components smaller, so they're not as intrusive on a regular day... When a couple of times the [SNP] got pointed out toward the outside of my leg I went through a doorway and whacked it on the doorway and they (the study engineers) had to look at it. Hope I didn't break it. (Interview 4)*

Introspection

During the interviews, the participant reflected on how his experiences with the SNP impacted his views on his abilities to use his prosthesis and his overall self-concept. These descriptions of his self-concept were coded in the "Introspection" node.

While working with and learning how to use the SNP, the participant reflected on how various portions of his personality or worldview influenced his experience with the device. For example, when asked if he was excited to take the SNP home for the first time, he notes that he is excited because it will be a challenge and that he was excited by challenges:

> *I like to be challenged. And doing the [sensory] exercises will be a challenge. Working on mentally connecting the things (sensations to prosthesis movements) will be a little bit of a challenge and stuff and trying to feel when it (the sensation) is there. (Interview1)*

In many instances, the participant described himself as a 'doer' who would take the steps necessary to complete a task or overcome a problem. In terms of completing daily tasks, he notes that he is generally very confident.

> *And I don't really doubt myself. I just go and do things. I'm fairly confident with what I do. (Interview 3)*

When reflecting on how he interacted with the sensation provided by the SNP, the participant noted the ease with which he could choose to ignore sensory stimuli or events that are unpleasant. This ability to ignore sensation, both related to his amputation and in general, was mentioned as a strategy he often used in his everyday life. Though he described himself as being able to ignore discomfort, he reflected that this capacity might have downsides.

> *I've always kind of ignored whatever was bothering me to get done whatever, what I was going to do …I just put it (discomfort or pain) out of my mind and do it…I can hit myself in the hand with a hammer when I am pounding on a punch and I don't have to miss a stroke I keep right on beating on the punch... I'm not so sure that it's a good mindset but it is the one I have. (Interview 5)*

<u>Familiarization with the System</u>

The "Familiarization with the System" node contains quotes where the participant remarked on experimenting with the device settings or learning how the device worked. Upon receiving the SNP, the participant first attempted to passively understand how the system delivered stimulation and the corresponding sensations. Then, he intentionally changed the stimulation settings to determine how these changes modified his sensory experience.

> *I'm starting to learn. At first, you know, it's kind of wearing [the SNP and] seeing what it did. Now I'm starting to play with stims (the stimulation) and studying [the sensation] a little bit more… I'm learning how to use it (the sensation). (Interview 2)*

Many quotes in this node detail the participant's process of changing the stimulation settings and learning what settings provide the best stimulation. The initial period of actively experimenting with the sensation led the participant to understand that lower levels of stimulation were more beneficial to him.

> *At the beginning, I was just kind of using [the SNP] all day and seeing how it affected me a little bit and stuff like that. And like I said, I had the wrong impression. I kept thinking I wanted [the stimulation as] strong as I could [get it]. (Interview 2)*

Although he was given at home exercises to concentrate on the sensation, he reported developing his own training strategies to familiarize himself with the SNP. He found that the best way for him to feel the sensations from the SNP was slowly rocking his prosthetic foot back and forth. This gave him the ability to notice how different movements would change his sensory experience from the SNP.

> *That's what I think that exercise I invented, rocking back and forth is doing. I can feel the transition from my toes to my midfoot to my heel back and forth like that. You know? I was impressed when I started doing it. It just made me think this is the right thing to do. (Interview 2)*

Own Prosthesis

The “Own prosthesis” node contains comments about the participant’s personal prosthesis that he owned and wore before beginning the SNP home study. His own prosthesis was retrofitted with the external SNP device components, such as the pressure sensors in the shoe, so that he could maintain his current socket fit and prosthesis alignment. Thus, some of the comments in this node may also pertain to experiences he had during the home study with the SNP. However, comments in this node are not directly related to SNP system components or capabilities.

Prior to the study the participant considered himself a proficient prosthesis user and was satisfied that his prosthesis did not require much maintenance. He spoke about his satisfaction with the fit and stability of his current prosthesis socket.

> *This is probably the best socket I've had out of over all the years…And I haven't had to have any adjustments made to it. (Interview 1)*

His satisfaction with his own prosthesis was so great, and he had so few problems with the device, that he felt that the prosthesis was a part of him. This also allowed him to use his own prosthesis without requiring focus or conscious thought.

> *You know, it's just a, it's almost a part of me now, in a way, you know? I just, I really don't pay any attention or notice it's (the prosthesis is) there unless there's a problem, and I don't run into that many problems most of the time. (Interview 1)*

When asked what factors of his own prosthesis are important to him, he stated that socket fit was the most important aspect, and the alignment of the foot relative to the pylon was the second most important aspect, since it impacts walking ability.

> *I think the biggest thing is the fit of the socket. [It’s] gotta be comfortable before you can stay at it (use the prosthesis consistently). The other thing is probably the alignment of the foot. You can't have it straightforward - It's hard to walk over the toe. (Interview 1)*

During the middle of the trial, the subject was fit for a new prosthesis by his clinical prosthetist. This fitting was unrelated to the SNP study and was part of the participant’s routine clinical care. He wanted to try a new type of prosthesis shank that has a shock absorbing feature. However, after being fit with the new shock absorbing prosthesis, he did not like being unable to feel the ground. He stated that he preferred a prosthesis with a rigid pylon due to its stability compared to a shock absorbing socket. Using the referred pressure from the ground helps him to maintain balance and walk when he is not receiving sensory feedback about foot-floor interactions from the SNP.

> *I don't like [the] rubber ball type socket (shock absorbing feature)... I don't feel it's stable... It's got a little squishy kind of movement, but I don't like it. I would rather feel the ground good and solid than to have a little bit of extra comfort that it provides. It is not enough comfort to be worth what I lose in the feel of the ground (Interview 5)*

Technical Aspects of the System

Descriptions about the process through which the participant donned and set-up the SNP system, as well as suggestions for future device improvements were captured in the "Technical Aspects of the System" node.

Each day the system would need to be donned and set up in a process that included selecting the stimulation levels for each channel. The participant described this as a quick process:

> *Oh, [the SNP system] doesn't take very long to put it on. I take maybe 5 minutes or something like that to put it on. Change the batteries real quick. Connect the cable up to my leg, and tape it down so it doesn't get pulled up. Then put the [external stimulator] on and plug it in. And program it to the [stimulation levels] that I want it on. (Interview 3)*

The participant frequently suggested improvements that could be made to the system. Many of these suggestions centered around miniaturizing the system, or "getting the components smaller." Other suggestions related to the complexity or durability of the system.

> *The biggest thing is to try at this point to maybe try to make the system a little more user friendly, and durable. (Interview 4)*

Appendix 2

Appendix 2 outlines a summary of the objectives and questions in each of the five semi-structured interviews. Spontaneous questions, follow-up questions, and probes asked in these interviews are not included.

**Interview 1: Baseline Interview**<br>***Objectives:***<br>To evaluate and record the participant's current point of view on their prosthesis, their expectations and goals for the study, and their phantom limb.
What are your goals for the study?
What are some of your concerns?
What are you most looking forward to doing once you take the SNP home?
What do you think is going to change about your daily life during the study?
What tasks to you want to do or try with the SNP?
Please describe your current prosthesis in as much detail as possible. (Socket, fit, features)
What types of tasks or movements are easy with your prosthesis?
What types of tasks or movements are difficult with your prosthesis?
Are there tasks or activities you choose not to do when wearing your prosthesis?
Describe your missing (phantom) limb?
Can you describe the movements you can make with your phantom limb?
How much concentration does it take to move or feel your phantom limb?
Does your phantom change when you wear a prosthesis?

**Interviews 2 and 3: Active Phase**<br>***Objectives:***<br>Discuss the experience of using the SNP outside of the lab, including general thoughts and satisfaction with the SNP and sensations. Evaluate the impact of the SNP on focus, confidence, sensation and the perceived phantom limb.
Can you tell me about your experiences using the SNP in your home and community?
How long did you have the SNP turned on each day?
What factors influenced your decision to turn on the SNP?
What did you think about the sensory feedback?
Did you change the way you used the prosthesis?
How satisfied were you with the SNP?
Describe any problems with the SNP?
How long did it take you to don the SNP, and how did it impact your routine?
In what situations did you need to re-calibrate the sensors or stimulation parameters?
What did the sensation feel like? Were there any changes over the course of using the device?
How much attention did you feel you needed to pay to the prosthesis when using the SNP?
What did the sensation tell you about where your prosthetic foot was when walking or doing a task?
Describe your phantom limb, were there any changes in the way you move or feel your phantom over the past week?

Interview 4 and 5: Post Active
Objectives: Discuss the experience of using the prosthesis without sensation outside of the lab. Compare the experience without sensation to the previous experience with sensation out of the lab. Discuss overall impressions of the take home study and expectations for the future.
Can you tell me about your experiences using the SNP without sensation in your home and community?
What kinds of tasks or activities did you do?
Were there any new tasks or activities that you did in the past two weeks that you hadn't attempted in the previous stages of the study?
What was it like to no longer have sensation after having it in the previous stage?
How did the lack of sensation change the way you viewed or thought about your prosthesis?
Compare your experiences with sensation to those without, did you prefer having sensory feedback or to not have sensory feedback?
Reflecting on the entire study, what happened as you expected? What was unexpected? What went well?
How did participating in this home trial impact your view of the overall research study?
How did your viewpoints about the value, benefits and limitations of sensory feedback change?
If there were an opportunity, would you want to use the SNP again in the future? Why or why not?
If you had the SNP to use all the time, how do you think it would integrate in with your current prosthesis.
Describe your phantom limb, were there any changes in the way you move or feel your phantom over the past week?
